# Re-crushing process and non-Darcian seepage characteristics of broken coal medium in coal mine water inrush

**DOI:** 10.1038/s41598-021-90449-3

**Published:** 2021-05-31

**Authors:** Mingkun Pang, Tianjun Zhang, Yi Guo, Lei Zhang

**Affiliations:** 1 College of Safety Science and Engineering, Xi’an University of Science and Technology, Xi’an, 710054 China; 2 Key Laboratory of Western Mine Exploitation and Hazard Prevention of the Ministry of Education, Xi’an, China

**Keywords:** Hydrology, Natural hazards

## Abstract

The initiation process of the mine water inrush accident, the essence of this process is the sudden change of the seepage state of the broken coal medium under pressure and the instability of the skeleton. In order to study the re-crushing mechanism and seepage characteristics of the broken coal medium under load, a set of three-axis seepage system was designed independently. Using the steady-state infiltration method, multiple flow factors under different particle size combinations and different stress conditions of the broken coal medium were obtained. The results of the study indicate: in one hand, the reduction of the porosity of the broken coal medium will cause the flow channel to be rebuilt, and the sudden change of flow rate will directly lead to the non-Darcian flow behavior. The early stage of compaction mainly affects the permeability *k* value, and the later stage of compaction mainly affects the non-Darcian *β* value; On the other hand, the seepage throat in the broken coal medium may have a sharp increase in its flow rate, leading to a sudden change in the flow pattern. The critical Reynolds number is also used to determine whether non-Darcian flow is formed, and its value in the water inrush system is about 40–133; at the same time, the non-Darcian flow in the broken coal medium conforms to the Forchheimer-type flow law. By analyzing the dependence relationship between factors, a seepage factor representation algebraic relationship suitable for Forchheimer type non-Darcian flow of broken coal medium is given, which can be used as a calculation basis in the prevention and treatment of mine water inrush accidents.

## Introduction

Coal mine water inrush is a serious mine accident, which is closely related to the flow state of the groundwater system^1,2^. A large number of scholars have shown that many groundwater systems are basically non-Darcian flows^3–5^. However, the non-Darcian seepage movement in the broken coal medium mainly changes with the change of flow velocity, which also leads to the deviation between the pressure gradient and the movement speed, and the fluid exhibits the non-Darcian flow characteristics^6,7^. Generally speaking, in the broken coal medium or porous coal medium, due to the deformation of the skeleton, the flow rate changes dramatically, and the fluid flow law does not follow Darcy’s law^8–10^.

Academic literature shows that the flow of groundwater only occurs in interconnected pores, and the size and shape of these porosities change with the change of applied stress^11,12^. By studying the flow characteristics of porous rock media under pressure, Ma et al.^13^ proposed a logarithmic equation between axial pressure and permeability coefficient. By studying the effect of external compressive stress on the seepage characteristics of granular coal, Ma et al.^14^ and Zhang et al.^15^ found that at higher compression rates, the strength of particle breakage is greater. As the porosity of the sample gradually decreased, Wu et al.^16^ and Ma et al.^17^ believed that particle breakage during compaction was the main reason for the generation of tiny particles, which would show a non-Darcian seepage state. From the perspective of the micro-inertia occurring in non-Darcian, Amitzur et al.^18^ believed that with the increase of flow velocity, local vortices and streamline bending will occur in the flow channel. He attributed the non-Darcian phenomenon to the tiny inertial force. Through extensive research on permeability factors, Ruth et al.^19^ believes that the Forchheimer number is a good factor to describe the occurrence of non-Darcian effect, and it is one of the key factors that determine the non-Darcian coefficient. Using the sand filling experiment method to analyze the fluid flow characteristics in porous media, Koeppel et al.^20^ showed the effect of non-Darcian flow on irregular materials of fracturing sand. By studying the inertial flow in porous media, Ren et al.^21^ believed that the *Forchheimer equation* and the *Izbash equation* can well describe the flow process in a larger velocity range.

The non-Darcian flow in the broken coal medium is related to coal particle size, pore pressure, seepage vector and other factors. Due to the particularity and importance of the non-Darcian effect, we need to determine when and where non-Darcian flow occurs in mines with water inrush risk, and we must determine the critical conversion point of the Darcy flow to the non-Darcian flow. Through the self-designed three axials seepage test system, the seepage test was carried out on porous coal samples with different initial porosity. In the experiment, the flow state of non-Darcian behavior was measured, and the hydraulic characteristics of non-Darcian flow were given; and the critical Reynolds number of non-Darcian flow was analyzed; and the factor change law was obtained in non-Darcian seepage process. These research conclusions can provide a certain theoretical guiding significance for mine water hazard management.

## Non-Darcian theory

Fluid flows in porous media, when the seepage velocity increases to a certain limit, there is no longer a linear relationship between the seepage velocity and the pressure gradient. For the broken coal medium system, this phenomenon is called non-Darcian infiltration behavior. At the same time, the system is also accompanied by huge changes in some factors. The following are the core factors to determine the occurrence of non-Darcian behavior: Grain porosity, Reynolds number, Forchheimer number and so on. Next, we will focus on the discussion of the above three core factors.

### Porosity under non-Darcian flow

We use *v*_i_ to indicate the flow velocity in the porous medium and *φ* to indicate the porosity. In a broken coal medium system, for porous materials composed of broken coal particles^22^, the Forchheimer equation can be written:

1 $$\rho_{0} \left( {\frac{1}{\phi }v_{i,t} + \frac{{1.75d_{g} }}{150}\frac{172.8}{{d_{g}^{2} }}\frac{{\left( {1 - \phi } \right)}}{{\phi^{3} }}\left| {\mathbf{v}} \right|v_{i} } \right) = - \mu \frac{172.8}{{d_{g}^{2} }}\frac{{\left( {1 - \phi } \right)^{2} }}{{\phi^{3} }}v_{i} - p_{i}$$

where *q*_0_, *l*, and *p* are constant fluid density, dynamic viscosity, and pressure, respectively. *d*_g_ is the diameter of crystal grains composed of a skeleton of porous material. In fact the terms ${{\left( {1.75d_{g} } \right)} \mathord{\left/ {\vphantom {{\left( {1.75d_{g} } \right)} {150}}} \right. \kern-\nulldelimiterspace} {150}}$ and ${{172.8} \mathord{\left/ {\vphantom {{172.8} {d_{g}^{2} }}} \right. \kern-\nulldelimiterspace} {d_{g}^{2} }}$ represent geometric factors describing the relationship between the skeletal material and the Forchheimer coefficient $\left| {\mathbf{v}} \right|v_{i}$, and the Darcy coefficient.

2 $$\frac{172.8\mu }{{d_{g}^{2} }}\frac{{\left( {1 - \phi } \right)^{2} }}{{\phi^{3} }} = \frac{\mu }{K}$$

where K is the permeability. If the solid part of the material is not composed of spheres then equivalent geometrical factors may be used. Nield and Bejan^22^ present an argument based on a porous medium of parallel tubes of uniform cross section to show that the coefficient $\rho_{0} \phi^{ - 1}$ should be replaced by ${{r\left( {1 - \phi } \right)^{2} } \mathord{\left/ {\vphantom {{r\left( {1 - \phi } \right)^{2} } {\phi^{3} }}} \right. \kern-\nulldelimiterspace} {\phi^{3} }}$ for some constant *r*.

We have seen that the porosity dependence of the Darcy coefficient is important. However, given that there is some controversy over the form of the inertia coefficient. ${{\left( {\rho_{0} v_{i,t} } \right)} \mathord{\left/ {\vphantom {{\left( {\rho_{0} v_{i,t} } \right)} \phi }} \right. \kern-\nulldelimiterspace} \phi }$ might not be general enough for most porous materials, Lyubimov et al.^23^ gives more general expression for the coefficients of the linear *v*_i_ term and of the Forchheimer, $\left| {\mathbf{v}} \right|v_{i}$, term.

3 $$u_{i,t} + bf(\phi )\left| {\mathbf{u}} \right|u_{i} = - \lambda h(\phi )u_{i} - \frac{l(\phi )}{{\rho 0}}p_{i}$$

The relationship can easily solve its result. However, we use the intermediate value theorem in the form:

4 $$f(\phi_{1} ) - f(\phi_{2} ) = (\phi_{1} - \phi_{2} )f^{\prime}(\xi )$$

For some $\phi_{1} < \xi < \phi_{2}$, One then has simply to check that $f^{\prime}$ is suitably bounded, mutatis mutandis. This allows us to have far more general forms for the functional dependence of $\phi$ for the coefficients.

### Non-Darcian critical Reynolds number

High-speed nonlinear flow is considered to be similar to the turbulent flow in the pipeline. The Reynolds number is used to describe the non-Darcian flow in the porous medium to identify the turbulent flow in the pipeline. Chilton et al.^24^ also conducted seepage experiments on filled particles, defining the Reynolds number as:

5 $$R{\text{e}} = {{\left( {\rho D_{{\text{p}}} v} \right)} \mathord{\left/ {\vphantom {{\left( {\rho D_{{\text{p}}} v} \right)} \mu }} \right. \kern-\nulldelimiterspace} \mu }$$

where *D*_p_ is the diameter of the particles. Chilton's experiments showed that the critical Reynolds number for a non-Darcian flow is larger than the others.

The Reynolds number has also been defined as follows^25^. The critical Reynolds number for a high velocity nonlinear flow is smaller than the others.

6 $$R{\text{e}} = {{\left( {\rho lv} \right)} \mathord{\left/ {\vphantom {{\left( {\rho lv} \right)} \mu }} \right. \kern-\nulldelimiterspace} \mu }$$

where *ρ* is the fluid density, *μ* is the fluid viscosity, *l* is the characteristic length, and *v* is the macroscopic velocity.

The factor $\sqrt k$ has been proposed to replace the particle diameter in the above equation, as follows^26^:

7 $$R{\text{e}} = \frac{10}{{\phi^{2.3} }}\frac{\rho v\sqrt k }{\mu }$$

The factor $\sqrt {{k \mathord{\left/ {\vphantom {k \phi }} \right. \kern-\nulldelimiterspace} \phi }}$ has also been proposed instead of the particle diameter, and the Reynolds number was defined as follows^27^:

8 $$R{\text{e}} = \frac{\rho v}{\mu }\sqrt {\frac{k}{\phi }}$$

where the critical Reynolds number is over the range of 0.022–0.29.

### Forchheimer number *F*o

The Forchheimer number has been proposed as the critical factor to estimate the conversion from Darcy flow to non-Darcian flow, and it is expressed as follows^28^:

9 $$F{\text{o = }}\frac{k\beta \rho v}{\mu }$$

The ratio of the fluid inertia pressure gradient and the viscous resistance pressure gradient is the Forchheimer number. Therefore, the Forchheimer number is the ratio of inertial resistance and viscous resistance.

After studying a large number of non-Darcian effects, Didier et al.^29^ defined the total pressure gradient consumed in overcoming the interaction between liquid and solid as the non-Darcian effect *E*, whose expression is:

10 $$E = \frac{{\rho \beta v^{2} }}{{{{ - dp} \mathord{\left/ {\vphantom {{ - dp} {dx}}} \right. \kern-\nulldelimiterspace} {dx}}}}$$

The non-Darcian effect *E* is a number related to the Forchheimer number, through the following relationship:

11 $$E_{c} = \frac{Fo}{{1 + Fo}}$$

Therefore, the ratio *E*_c_ is called non-Darcian error, that is, the effect of non-Darcian effect on the result is ignored. This relationship can be applied to numerical simulations to determine whether non-Darcian effects are considered in the model. To use the critical Forchheimer number, the above formula can also be rewritten as:

12 $$Fo_{c} = \frac{{E_{c} }}{{1 - E_{c} }}$$

This formula is based on the expression of non-Darcian effect. This value can define the corresponding critical Forchheimer number according to the characteristics of the specific problem. Didier et al.^29^ believe that when its value changes around 0.11, it is equivalent to 10% of non-Darcian effect.

## Experiments

After a long period of deposition, compaction, and re-cementing, broken coal will form a special porous medium, most of which are coarse-grained materials. When the hydraulic gradient is large and the flow rate is high, it often shows non-Darcian Seepage state. Meanwhile, the permeability of porous media is mainly affected by the stress rate^30^, Deformation of skeletal structure, both presence of water and magnitude of water saturation^31,32^. In order to capture the compaction and deformation characteristics of coal samples of different particle sizes. In this paper, through the independent design of the three axials seepage system, the steady-state seepage method was used to study the flow characteristics of the broken coal with mixed particles, and quantification of the effect of changing grain sizes and axial displacements on the properties of water flow in a non-Darcian condition. In the following sections, the details of test specimens and testing, experimental apparatus, and procedure are explained.

### Materials

#### Material preparation

Considering that different gradation structures have different pore characteristics, the sample aggregate prepared in this paper includes four sizes, and the mass distribution within each particle size range is subject to *Talbot theory*^33^, namely:

13 $$P_{i} = \left( {{{d_{i} } \mathord{\left/ {\vphantom {{d_{i} } D}} \right. \kern-\nulldelimiterspace} D}} \right)^{n} \times 100\%$$

**Figure 1:**
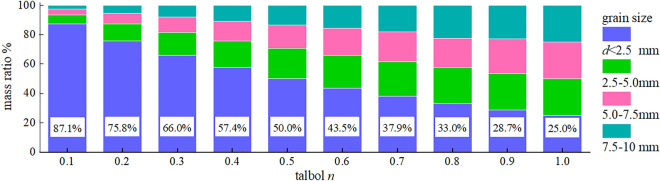
The ratio of particle size in the sample.

In the formula: *P*_*i*_ is the ratio of aggregate particles with diameter less than or equal to *d*_i_. *D* is the maximum diameter of aggregate particles; *n* is the Talbot index; as the value of *n* increases, the content of large particles in the aggregate increases. Calculated by Talbot's theoretical formula, the mass distribution of aggregate particles in each size range is shown in Fig. 1.

#### Experimental procedure

**Figure 2:**
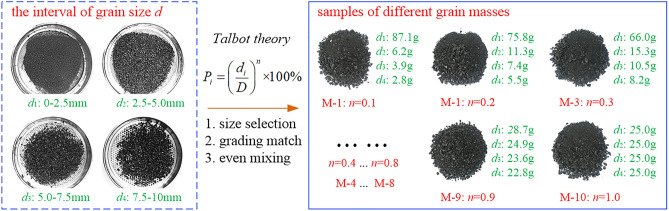
Sample preparation process.

The coal samples used in the test were taken from CUIJIAGOU Coal Mine, Shaanxi Province. First, the broken coal sample is selected, and four particle sizes are selected for gradation. The test aggregates are *d*_1_ (0–2.5 mm), particle size *d*_2_ (2.5–5.0 mm), particle size *d*_3_ (5.0–7.5 mm), and particle size *d*_4_ (7.5–10 mm). According to the rock mechanics test requirements, that is, the ratio of the sample diameter *D* to the maximum particle diameter *d* should satisfy *D*/*d* ≥ 5, so the maximum particle size of this test is selected to be 10 mm. Next, the ratio of broken coal with different particle sizes is adjusted. According to the *Theory of Continuous Gradation* of broken coal rock mass^34^, Talbot index *n* takes 10 sets of samples in sequence from 0.1, 0.2 to 1.0, and numbers them M-1, M-2 to M-10. Finally, the DDL600 rock mechanics test system is combined with a self-made three axials permeameter (XUST700) to conduct a seepage test on the broken coal. The sample preparation process is shown in Fig. 2.

### Methodologies

#### Experiment design

**Figure 3:**
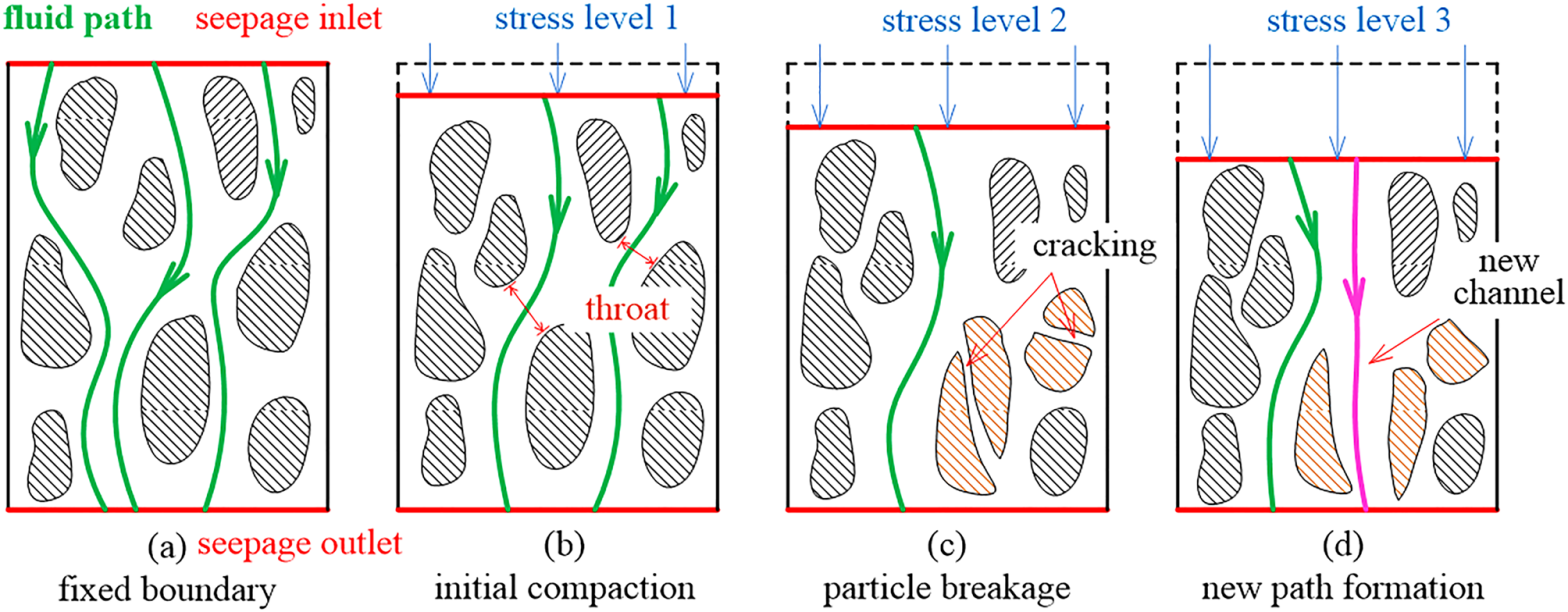
Structural deformation diagram.

According to the test design, under graded loading, seepage tests of broken coals with different gradation structures are carried out, and 30 grade loads (from 1 to 30 KN in increments of 1 KN) are applied axially, providing 30 grade stress conditions, they are denoted as *stress 1*, *stress 2*, …, *stress 30*. Set 10 levels of osmotic pressure (from 0.2 to 2.0 MPa in increments of 0.2 MPa) under each axial load, and provide ten levels of osmotic pressure, which are denoted as *seepage 1, seepage 2, …, seepage 10*. Among them, the data of axial load *F*, axial displacement $\Delta h$ and loading time *t* are all recorded by computer. A diagram of the deformation process of the broken coal medium is shown in Fig. 3.

The mechanical deformation of broken coal medium refers to the phenomenon that broken coal particles are compacted and densified under high pressure pore-water. Under the condition of high stress, the porosity decreases sharply, and the coal particle is further broken. In the end, two possibilities coexist: one possibility is that the inner part of the sample is closed and the permeability is reduced; the other possibility is that the particles inside the sample readjust their posture and new channels are generated.

#### Experimental equipment

**Figure 4:**
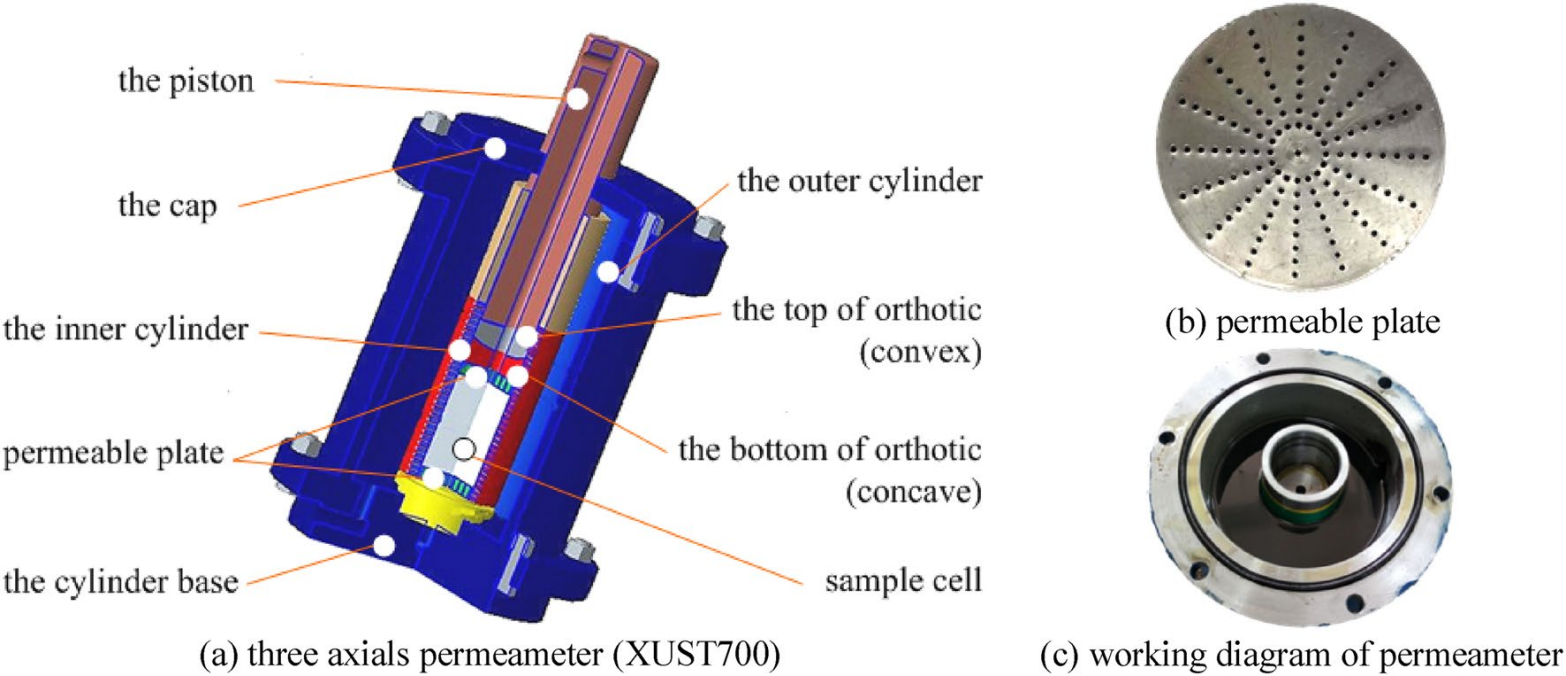
Sample installation diagram.

In order to provide such special seepage mechanical conditions, a set of three-axis seepage test system (XUST700) was made, which mainly includes: axial pressure control part, osmotic pressure control part, data acquisition part; The system can achieve axial pressure range (0–600 KN), seepage pressure range (0–10 MPa), lateral pressure range (0–5 MPa) et al. Sample size:*φ*50 × 50 mm, *φ*50 × 75 mm, *φ*50 × 100 mm and other sizes samples; Sample materials can meet: natural core, natural coal, artificial samples and other material samples; Permeate is optional: DTE22 hydraulic oil (*μ* = 1.96 × 10^–2^ Pa s, *ρ* = 874 kg/m^3^), water (*μ* = 100.2 × 10^–5^ Pa s, *ρ* = 988.2 kg/m^3^). The Sample installation diagram is shown in Fig. 4.

### Ethical approval

All research in the manuscript complies with ethical requirements and consent to participate. Finally, thanks to the test platform provided by Key Laboratory of Western Mine Exploitation and Hazard Prevention of the Ministry of Education, the test was successfully completed and the data was obtained.

## Results and discussions

The key to determining the non-Darcian effect is the accurate determination of factors in the seepage process, such as: porosity, permeability, Reynolds number, and Forchheimer number. By analyzing the overall characteristics and dependencies of these factors, the mechanism of water inrush accidents can be explained.

### Behavior distribution of non-Darcian flow

When the porosity is large, there are many pore channels inside the sample, and the flow velocity is large, which basically conforms to the Darcy linear pattern; while when the porosity is small, the pore channels inside the sample are closed, the flow velocity is small, and the deviation is in a non-Darcian state. For linear seepage, Darcy summed up the well-known Darcy's law of porous media penetration based on the water penetration test in the sand column in 1856:

14 $$v = - \frac{{k_{D} }}{\mu }\frac{\partial p}{{\partial x}}$$

In the formula, *v* is the flow rate; *k*_D_ is the permeability corresponding to the Darcy flow; *μ* is the dynamic viscosity of the fluid; *p* is the water pressure; *x* is the height.

On the one hand, if the seepage velocity changes drastically, the flow state will not fully comply with the linear law, and thus non-Darcian flow will occur; the Izbash type non-Darcian seepage equation is:

15 $$v^{n} = - \frac{{k_{e} }}{\mu }\frac{\partial p}{{\partial l}}$$

In the formula, *v* is the flow rate; *n* is the coefficient of the Izbash equation, and 1 ≤ n ≤ 2, *k*_e_ is the permeability; *μ* is the dynamic viscosity of the fluid; *p* is the water pressure; *l* is the length of the flow path.

On the other hand, the fluid (gas) seeps at a higher velocity. Due to the effects of inertial force and turbulence, it usually exhibits the non-Darcian law; the low-speed Forchheimer non-Darcian seepage equation considering the starting pressure gradient is as follows:

16 $$- G_{p} = \frac{\mu }{k}v + \beta \rho v^{2}$$

**Figure 5:**
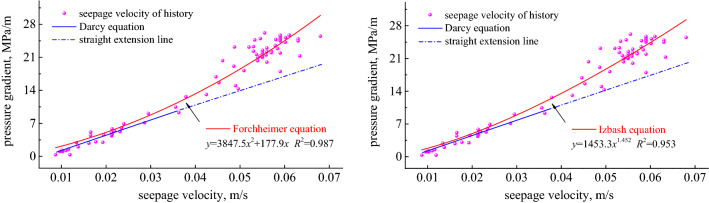
Comparison of historical data with the calculated result of the Darcy and non-Darcian flow patterns.

In the formula, *G*_*p*_ is the pore pressure gradient; *μ* is the dynamic viscosity of the fluid; *k* is the permeability corresponding to the non-Darcian flow, *v* is the flow rate; *β* is the non-Darcian flow coefficient; *ρ* is the fluid density. The relationship between pressure gradient and velocity is shown in Fig. 5.

We tested ten sets of samples in the laboratory using a three-axis permeameter, and the result statistics are on the coordinate axis, the horizontal axis represents the penetration velocity, and the vertical axis represents the pressure gradient. The figure shows that when the flow rate is relatively low, the pressure gradient and the flow rate have a good linear relationship; as the seepage velocity increases, the curve shows a slope that deviates from the linear rule, gradually approaching the pressure gradient axis, showing a significant non-linear Darcy phenomenon. If we follow the Darcy’s equation, we get *k*_D_ = 5.09 μm^2^. According to Forchheimer's non-Darcian seepage equation, through factor fitting, the deviation factor *β* is 1.96 × 10^10^/m, and *k* is 4.91 μm^2^, its fitted correlation coefficient *R*^2^ was 0.987. This calculated value of permeability is smaller than *k*_D_. According to the Izbash non-Darcian seepage equation, through factor fitting, the equation coefficient *n* is 1.452 and *k*_*e*_ is 6.01 μm^2^, its fitted correlation coefficient *R*^2^ was 0.953. This calculated value of permeability is larger than *k*_D_. For the broken coal system, the flow rate in porous media is generally in a high-speed flow state, which shows that the Forchheimer binomial equation can be used as a motion equation to describe the fluid flow of the mine water inrush system.

### The hydraulic characteristics of the non-Darcian flow

#### Calculate the initial porosity

Immerse a sample of a certain mass *m* in water for a certain period of time to make it initially saturated. At this time, the initial volume before broken can be calculated from the sample particle density *ρ*, $V_{0} = {m \mathord{\left/ {\vphantom {m \rho }} \right. \kern-\nulldelimiterspace} \rho }$. After connecting the penetrometer, load the sample into the cylinder and record the initial height *h*_0_ of the sample in the cylinder and the inner diameter *D* of the cylinder to obtain the initial volume $V_{0}^{^{\prime}}$ and initial porosity $\phi_{0}$ of the sample, calculated as follows:

17 $$V_{0}^{\prime } = \frac{{\pi D^{2} }}{4} \cdot h_{0} ,\quad \phi_{0} = \frac{{V_{0}^{\prime } - V_{0} }}{{V_{0}^{\prime } }}$$

#### Calculate the penetration factors

After completing the installation of the osmometer, load the initial pressure 1KN, the pressure head contacts the osmometer piston; start the water circulation, and let the sample water saturate again. Load the set pressure scheme, and keep the displacement *S* unchanged, and gradually realize the multi-level penetration pressure penetration test. The analysis can obtain a permeation factor under a stable pressure gradient. The upper pressure *p*_0_ and the lower pressure *p*_1_ of the test indicate that the value of the pressure gradient is:

18 $$\left. {\frac{\partial p}{{\partial x}}} \right|_{S} = - \frac{{p_{1} }}{H}$$

Among them, the sample height $H = h_{0} - S$. At the same time, the porosity of the sample under each axial pressure is:

19 $$\phi { = }1 - \frac{m}{{\rho A\left( {h_{0} - S} \right)}}$$

Through linear regression of pressure gradient and velocity $\left( {\left. {\frac{\partial p}{{\partial x}}} \right|_{S} - v} \right)$, the non-Darcian permeability factors *k* and *β* can be obtained.

#### Obtain the dependency relationship

**Figure 6:**
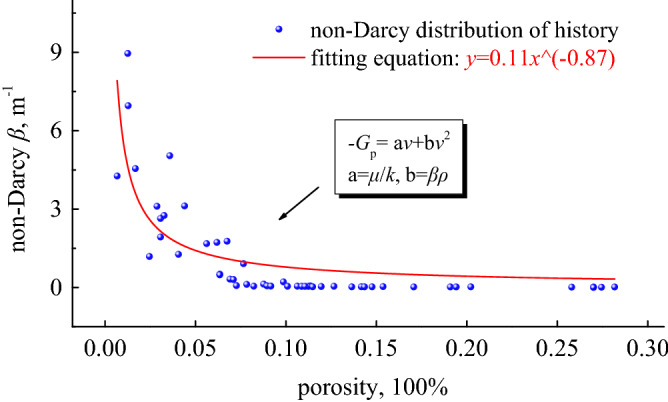
Non-Darcian factor and porosity curve.

**Figure 7:**
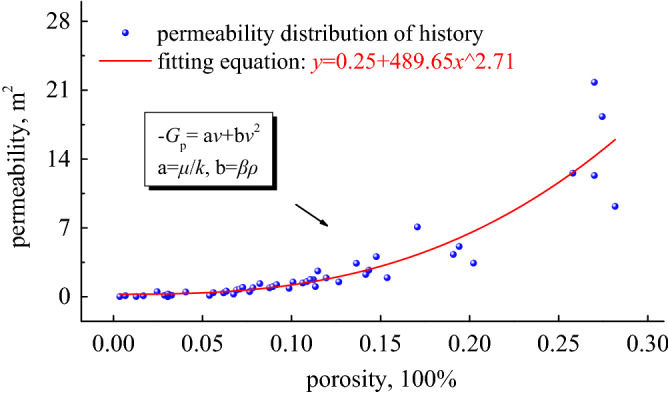
Permeability and porosity curves.

Gradually apply axial displacements of other levels to obtain *k* and *β* at each level of porosity $\phi$, draw scatter plots of $k - \phi$ and $\beta - \phi$ in the coordinate system, and obtain the functional relationship of $k = f\left( \phi \right)$ and $\beta = f\left( \phi \right)$ through regression analysis. The relationship between $\phi$ and *β* is shown in Fig. 6, and the relationship between $\phi$ and *k* is shown in Fig. 7.

We counted the test factors of all samples and displayed a large number of factor points on the coordinate axis, including the dependence curve of $\beta - \phi$ and the dependence curve of $k - \phi$. The figure shows that the effect of porosity on *β* value basically conforms to the change of negative exponential function law, and the effect of porosity on *k* value basically conforms to the change of exponential function law. In the initial stage of loading, due to the sharp decrease in the porosity of the bulk coal sample, the large amount of closing of the flow channel causes the permeability *k* to decrease sharply, but the *β* value does not change significantly. Generally speaking, after the coal sample is loaded, the porous medium skeleton deforms, and the pore structure may increase or slightly increase. These phenomena are closely related to the dislocation, breakage and shedding of grains in the porous medium, and depend on the number of internal effective seepage channels.

### Analysis of critical Reynolds number

According to the definition of fluid mechanics, Reynolds number is an important index to judge the motion state of viscous fluid, where *R*e_c_ represents the lower critical Reynolds number and *R*e′_c_ represents the upper critical Reynolds number.

When *R*e < *R*e_c_, the fluid flow state is laminar flow; when *R*e > *R*e′_c_, the fluid flow state is turbulent flow; when *R*e_c_ < *R*e < *R*e′_c_, laminar flow and turbulent flow coexist, and the flow state is extremely unstable If there is disturbance of external force, the laminar flow may change into turbulent state instantly. The calculation formula of Reynolds number is:

20 $$Re = \frac{\rho v\delta }{{\phi \mu }}$$

where, *ρ* is the density of the fluid, kg/m^3^; *v* is the average velocity of liquid penetration, m/s; *μ* is the kinematic viscosity of the fluid, m^2^/s; *δ* is the equivalent diameter of the flow channel, m.

For the flow of fluid in porous media, the resistance coefficient *f* is a function of the Reynolds number *R*e, for example: *f* = *F*(*R*e), the data can be dimensionless, and the relationship can be expressed as^35^:

21 $$f = \delta \frac{\Delta p}{{\rho \Delta l}}\left( {\frac{\phi A}{q}} \right)^{2}$$

here, *δ* is the characteristic length of the porous medium, and there is a relationship $\delta = \left( {{k \mathord{\left/ {\vphantom {k \phi }} \right. \kern-\nulldelimiterspace} \phi }} \right)^{0.5}$.

**Figure 8:**
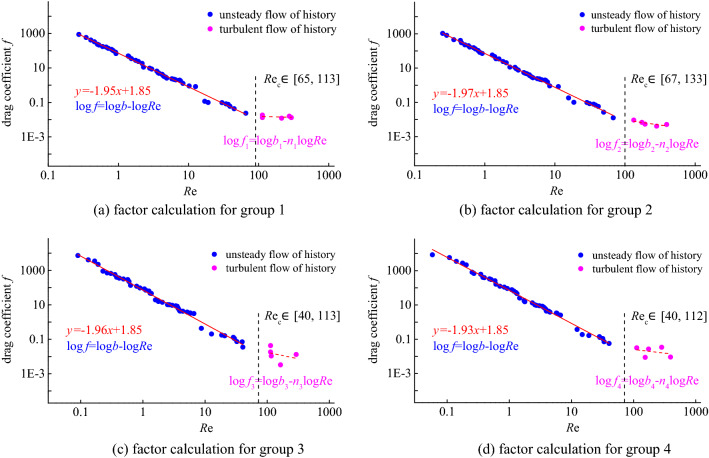
Curve of the resistance coefficient versus the Reynolds number.

By inserting the measured data (flow rate and pressure gradient) through the above formula, the factors of the fluid and porous medium can be obtained, including: calculating the resistance coefficient and calculating the corresponding Reynolds number. In the double logarithmic coordinates, draw the curve of Reynolds number and drag coefficient, repeat the design of the sample process four times, and mark it as four groups, the curve relationship is shown in Fig. 8.

By calculating the corresponding resistance coefficient value *f* and Reynolds value *R*e, their relationship curve is plotted on the double logarithmic coordinate axis. According to the analysis curve, the relationship between the drag coefficient and the Reynolds number can be divided into two sections: the first section, the flow rate is small, and the Reynolds number is also small, the two factors maintain a linear relationship, showing the Darcy seepage law; the second In the section, the flow velocity becomes larger and the Reynolds number is also larger. The two factors deviate from the linear trend, indicating the non-Darcian seepage stage. This indicates that there is a transition boundary for fluid flow transition from Darcy flow to non-Darcian flow. Correspondingly, there is a critical Reynolds number range suitable for broken coal system. The range is about 40–133 through statistics.

### Characterization of non-Darcian flow

For broken coal medium, its seepage state is related to the properties of porous media and seepage conditions. In the process of studying non-Darcian seepage, not only the influence of fluid properties but also the deformation law of porous media must be considered. According to a series of seepage tests, through fitting the pressure gradient and flow rate, Yao and Ge^35^ determined that the expression of non-Darcian coefficient *β* is:

22 $$\beta = \frac{a}{{k^{c} \phi^{b} }}$$

In order to conform to the dimension of Forchheimer-type non-Darcian seepage coefficient *β*, the value of *c* here should be 0.5, therefore $\beta = {a \mathord{\left/ {\vphantom {a {k^{c} \phi^{b} }}} \right. \kern-\nulldelimiterspace} {k^{c} \phi^{b} }}$.

23 $$\beta k^{0.5} = \frac{a}{{\phi^{b} }}$$

Taking logarithms on both sides of the above formula, it is clear that $\lg \beta k^{0.5}$ and $\lg \phi$ are linear. Therefore, linear regression processing can be performed on the relationship between *f* = F(*k*) to obtain the corresponding coefficients. The fitting results are analyzed and further transformed to determine the relationship between non-Darcian coefficient *β* and permeability, calculated as follows:

24 $$\lg \left( {\beta k^{0.5} /10^{6} } \right) = \lg \phi^{ - 2.5} + \lg 4112$$

**Figure 9:**
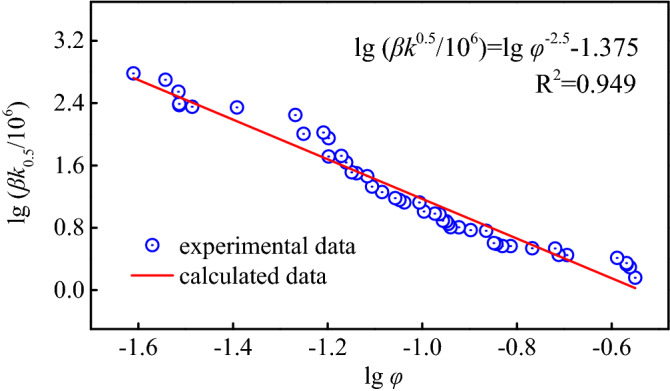
Non-Darcian coefficient of each sample through the fitting curve of the pressure gradient versus the flow rate.

Further simplified to:

25 $$\beta = \frac{{4.112 \times 10^{2} }}{{k^{0.5} \phi^{2.5} }}$$

here, k is the permeability, mm^2^; *β* is the non-Darcian coefficient, /cm. This relationship can be used to calculate the non-Darcian *β* value from the characteristic factors of coal particles, and the derivation process is reasonable. The non-Darcian coefficient of each sample is shown in Fig. 9.

The results show that under high stress conditions, in the compacted broken coal sample, the grains will break further, causing the internal pore channels of the sample to close, the flow rate will change suddenly, and non-Darcian behavior will occur. For the flow state description problem in the mine water inrush seepage system, the Forchheimer-type non-Darcian seepage equation is a good formula. Secondly, through derivation, we also obtained the relationship between the seepage factors of Forchheimer type non-Darcian flow suitable for broken coal system. The algebraic relationship between non-Darcian *β* and permeability *k* is given by fitting, and the results can be used to estimate the seepage factors.

In general, when non-Darcian flow occurs in the mine water inrush system, the flow state of the coal particle porous medium is easily abruptly changed by small changes in external stress; the action of high-pressure water may cause the large particles inside the porous medium to move Even if some small particles are eroded, the infiltration skeleton is extremely unstable at this time; a sharp increase in the porosity factor will cause an increase in the flow rate, and the infiltration will be converted into a pipeline flow, which may eventually induce a mine water inrush accident.

## Conclusions

By independently designing the three axials seepage test steel cylinder, the broken coal samples with different pore framework structures were studied. The judgment conditions, flow characteristics, etc. of non-Darcian seepage occurred in the broken coal medium system are given, and the mathematical model for calculating the flow factors is given. The conclusions are as follows.

1. The porosity factor of the broken coal medium is a sensitive factor that affects the change of infiltration factors. The change of porosity will directly lead to the reconstruction of the seepage channel, so that the sudden change of flow rate shows the behavior of non-Darcian flow. In the early stage of compaction, the permeability is significantly changed by the decrease of porosity ($k - \phi$); in the later stage of compaction, non-Darcian *β* is significantly affected by the porosity ($\beta - \phi$).
2. By introducing the drag coefficient, the critical Reynolds number of non-Darcian flow in the broken coal medium can be calculated. The Reynolds number is a very useful factor to determine the flow state of the fluid. The change of the seepage channel in the porous medium will cause a sharp increase in the flow velocity in the roar channel. Eventually, the fluid will appear similar to the turbulent flow in the pipeline. This critical Reynolds number has a value range of about 40–133.
3. Forchheimer-type non-Darcian flow factors have corresponding algebraic characterization relationships. The broken coal grains in the non-Darcian infiltration stage have been extremely dense, and the coal particles are mainly point-contacted, which first forms the form of tensile stress failure and is then further compacted. By calculating the non-Darcian coefficient in each test flow state, the seepage factors of Forchheimer-type non-Darcian flow in broken coal medium can be characterized as: $\beta = \frac{{4.112 \times 10^{2} }}{{k^{0.5} \phi^{2.5} }}$.

## Data Availability

The data used to support the findings of this study are available from the corresponding author upon request.
